# Supplementary material for: Gate-tunable quantum pathways of high harmonic generation in graphene
Source: Nat Commun. 2022 Nov 4;13:6630. doi: 10.1038/s41467-022-34337-y (PMC9636431; doi:10.1038/s41467-022-34337-y)
Supplement: Supplementary file 1 — Supplementary Information [file 41467_2022_34337_MOESM1_ESM.pdf]

Supplementary Information for

Gate-tunable quantum pathways of high-harmonic generation in graphene

Contents

Supplementary Figures (Figure S1 – S8)

Supplementary Note

Device fabrication

Infrared transmission spectroscopy

HHG measurements

Transition dipole moment of gapless Dirac material

Quantum master equation

Liouville-von Neumann equation

Dephasing parameter

Calculation of chemical potential dependent HHG

I_{exc} - dependence of the fifth harmonic expected from perturbative nonlinear optics

Discussion on detailed physical origin and features of μ-dependent HHG

Supplementary Figures

Figure S1. Elimination of background luminescence from emission spectrum on graphene

Fig. S1 displays emission spectrum on graphene illuminated by mid-infrared excitation. Total emission spectrum (black curve) features a broadband background signal in addition to the fifth and seventh harmonics at 1.38 eV and 1.93 eV, respectively. No broad background is detected from sapphire substrate, confirming that the broad background comes from graphene. The origin of the background is presumed to be luminescence from an impurity or defect in graphene[1], or gray-body radiation of hot electrons with elevated temperature of ~ 3000 K[2]. For all experimental data shown in manuscript, the contribution of the luminescence has been eliminated by subtracting polynomial fitting of broad background (red curve).

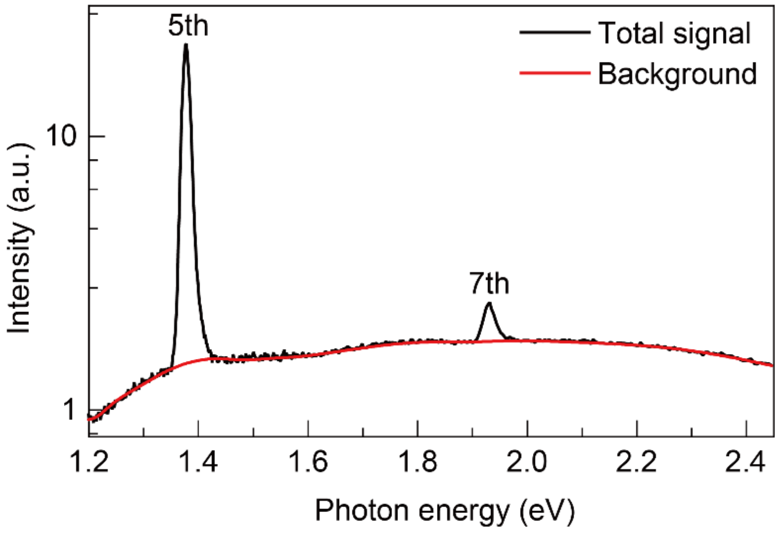

Figure. S1| Emission spectrum and background luminescence of graphene excited by mid-infrared pulses. The total emission spectrum of graphene (black curve) contains a luminescence background signal (red curve), in addition to the fifth (1.38 eV) and seventh harmonics (1.93 eV). We measure spectrum under laser excitation energy of 0.28 eV with I_{exc} = 50 GW cm^{-2}. To avoid any optical damage by intense mid-infrared excitation, optical shutter is synchronized with detector. The signal is acquired with illumination time of 100 ms.

Figure S2. Laser-induced damage of graphene by intense mid-infrared pulse

When the graphene is illuminated by intense mid-infrared pulse, an irreversible damage can be created on the exposed area, which is possibly due to the laser-induced local oxidations. To monitor the damage, we have checked the transient attenuation of fifth harmonic intensity for varying I_{exc}. As shown in Fig. S2a, the harmonic responses are accumulated in the unit exposure time of 100 ms. During this measurement, motorized optical shutter is applied to precisely control the exposure time of intense mid-infrared pulse. The harmonic signal exhibits constant amount for multiple measurement up to I_{exc} of 52.1 GW cm^{-2}, and attenuates at 109 GW cm^{-2}. The instantaneous attenuation refers to immediate damage under strong laser illumination. To avoid the contribution of this instantaneous damage, we have kept I_{exc} lower than the damage threshold for all measurements. On the other hand, many degree-of-freedoms should be incorporated during systematic measurement on the harmonic response of graphene, including the laser ellipticity (ε_{exc}), chemical potential (μ), and HHG polarization angle (φ), which accompany large amount of data points and thus require long measurement time. During whole experiments, mid-infrared excitation must guarantee the absence of any signal degradation, including thermal damage from averaged power. Therefore, we investigate the long-term damage on graphene. Fig. S2b displays the attenuation of harmonic responses in graphene under continuous exposure to examine laser-induced long-term damage. Above certain peak intensities (~ 7 GW cm^{-2}), the harmonic intensity starts to gradually decrease, which indicates the optical damage on graphene. We estimate the origin of this long-term damage as the averaged power from continuous mid-infrared illumination. In order to avoid laser-induced long-term damages, experimental condition is kept at I_{exc} of 3.1 GW cm^{-2} during measurement of μ- and ε_{exc}-dependent HHG polarization (see Fig. 1h, Fig. 2c-e and Fig. 3). On the other hand, for the relatively simple measurement, which does not require long acquisition time, we have used a motorized optical shutter to expose graphene only for 100 ms for acquisition of each data point in order to avoid short- and long-term damages for I_{exc} up 50 GW cm^{-2} (Fig .1d,e,g).

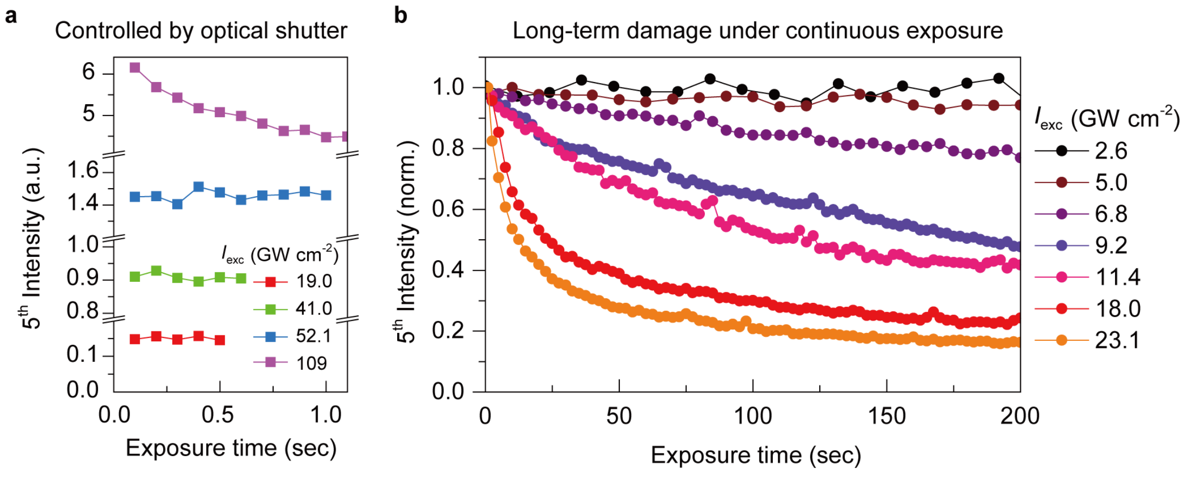

Figure. S2| Attenuation of the fifth harmonic signal under intense mid-infrared excitation. a, Harmonic responses accumulated in the unit exposure time of 100 ms for different I_{exc}. Harmonics are measured repeatedly to evaluate laser induced damage. It exhibits constant behavior up to I_{exc} of 52.1 GW cm^{-2}, but attenuates at 109 GW cm^{-2}. **b,** Harmonic responses for long-term measurement for various I_{exc}. The harmonic intensity is substantially attenuated during continuous measurement for I_{exc} above 7 GW cm^{-2}.

Figure S3. Sample orientation dependence of the fifth harmonics under linearly-polarized excitation

We perform crystal orientation dependence of fifth harmonics in graphene. Harmonic intensity is recorded under linearly-polarized excitation with I_{exc} of 3.1 GW cm^{-2} and E_{ph} of 0.28 eV. Instead of directly rotating the sample orientation, the polarization of incident pulse is rotated by half-wave plate placed before the sample. As shown in Fig. S3, we do not observe pronounced orientation dependence of fifth harmonics.

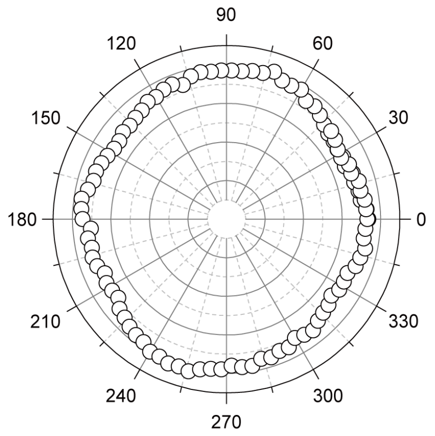

-

Figure. S3| Sample orientation dependence of the fifth harmonics under linearly-polarized excitation.

Figure S4. Photon energy dependence given by linearly-polarized excitation

We investigate the influence of E_{ph} on HHG to clarify a significance of multi-photon transition in HHG. During the measurements, excitation is given by linear polarization with I_{exc} of 3.1 GW cm^{-2}. Fig. S4a shows the fifth harmonic spectra with different E_{ph} from an additional device. Center photon energy of each fifth harmonic response exactly corresponds to $5E_{\mathrm{ph}}$ without any energy shift. As shown in Fig. S4b, μ dependence of the HHG for different E_{ph} exhibit similar resonance-like profiles. For E_{ph} of 0.28 eV, this device also shows the resonance-like profile with the maximum intensity at $2|\mu|$ = 0.94, which is similar to the device in the main text. On the other hand, for the same device and E_{ph} of 0.31 and 0.35 eV, harmonic intensity shows similar resonance-like profiles, but the entire profiles are precisely shifted with the laser excitation energies. As a function of $2|\mu|/E_{\mathrm{ph}}$, the resonance-like profiles for three different laser excitation energies overlaps each other in terms of the maximum intensity condition. Over different devices, we observe this condition varies from 3.1 to 3.6 as shown in Fig.S5.

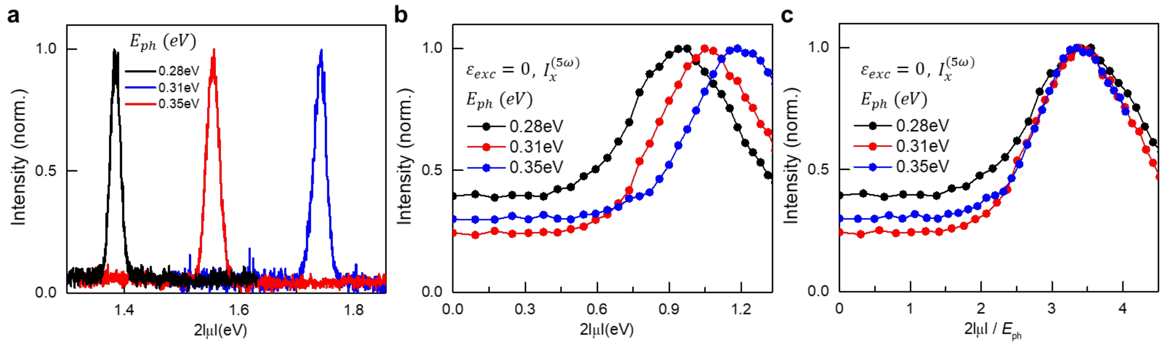

Figure. S4| Fifth harmonics under linearly-polarized laser with the different E_{ph}. a, Fifth harmonic spectra with E_{ph} of 0.28 eV(black line) 0.31 eV(red line) and 0.35 eV(blue line). **b,** Normalized harmonic intensity versus $2|\mu|$ with E_{ph} of 0.28 (black circles), 0.31 (red circles) and 0.35 eV (blue circles). **c**, Normalized $I_{\mathrm{x}}^{(5\omega)}$ versus $2|\mu|/E_{\mathrm{ph}}$ under linearly-polarized excitation for different E_{ph}.

Figure S5. Investigation of the empirical relation $2|\mu|/E_{ph}$

We characterize the position of $2|\mu|/E_{ph}$ at the maximum harmonic intensity for multiple samples and measurements to check reproducibility. Maximum peak position of $2|\mu|/E_{ph}$ is determined from zero-crossing point of harmonic intensity differentiated by μ. Fig. S5 shows the empirical relation of $2|\mu|/E_{ph}$ for multiple samples. We repeated the measurements 22 times, where $2|\mu|/E_{ph}$ ranges from 3.1 to 3.6, centered at the most common value of 3.4. Physical origin and variation of this empirical condition over devices are discussed in Supplementary Note (Discussion on detailed physical origin and features of μ-dependent HHG) below.

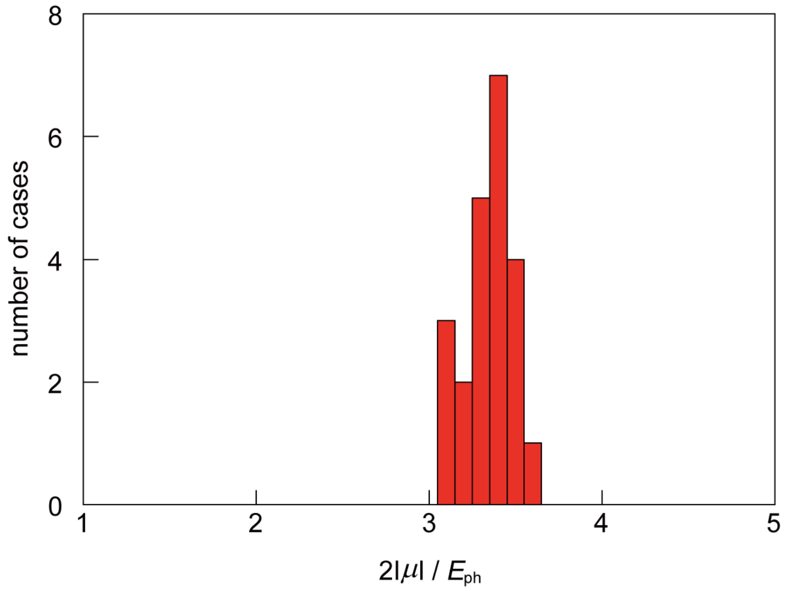

Figure. S5| Investigation of the peak position of μ-dependent harmonic response for multiple samples.

Figure S6. Sample orientation dependence under elliptically-polarized excitation

We now investigate sample orientation dependence with the fifth harmonics under elliptically-polarized excitation. We measure the fifth harmonic response with rotating the major axis of elliptical polarization ($\varepsilon_{\mathrm{exc}}$ = 0.3). Fig. S6a displays the excitation laser intensity profile. Each harmonic profile in Fig. S6b is acquired from elliptically-polarized excitation represented with the same color. All HHG polarization profile exhibit the same amount of the intensity and relative polarization rotation angle from the major axis of the excitation (25 degrees).

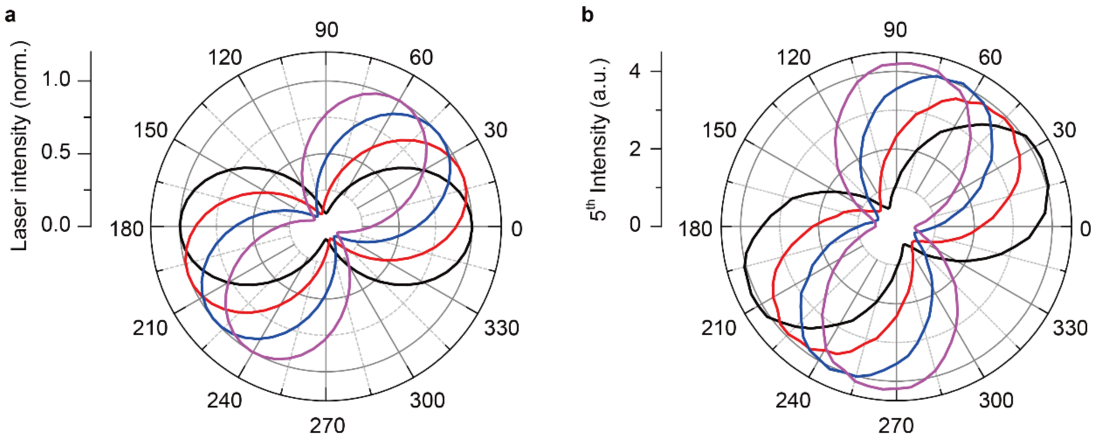

Figure. S6| Crystal orientation dependent fifth harmonics under elliptically-polarized excitation. **a,** Intensity profile of elliptically-polarized excitation laser ($\varepsilon_{\mathrm{exc}}$ = 0.3) with the rotating major axis. The major axis of excitation laser along the x-direction (black line) is rotated by 20 (red line), 40 (blue line), and 60 degrees (purple line). **b,** Polarization profile of harmonic signals measured under elliptically-polarized excitation shown in (a).

Figure S7. μ-dependent HHG under the elliptically-polarized excitation with different E_{ph}

Fig. S7 shows μ-dependent harmonic responses under elliptically-polarized excitation (ε_{exc} = 0.3) with different E_{ph} of 0.28, 0.31, and 0.35 eV. During the measurements, I_{exc} have kept at 3.1 GW cm^{-2}. We note that the measurement is carried out in the different device from the devices used for the main text and Fig.S4. However, observed phenomena below are consistent within the same device. As shown in Fig. S7a and b, $I_x^{(5\omega)}$ for all E_{ph} manifest resonance-like profiles similar to that obtained under the linearly-polarized excitation, satisfying empirical relation of $2|\mu|/E_{ph}$ ~ 3.1. In the case of $I_y^{(5\omega)}$ for varying E_{ph} (see Supplementary Fig. 7c), it exhibits step-like profiles similar to that obtained at E_{ph} = 0.28 eV. For both $I_x^{(5\omega)}$ and $I_y^{(5\omega)}$, their entire profiles are precisely shifted with the laser excitation energies as shown in Fig.S7a and S7c. As a function of $2|\mu|/E_{ph}$, $I_x^{(5\omega)}$ and $I_y^{(5\omega)}$ profiles overlap each other with an excellent agreement (Fig.S7b and S7d).

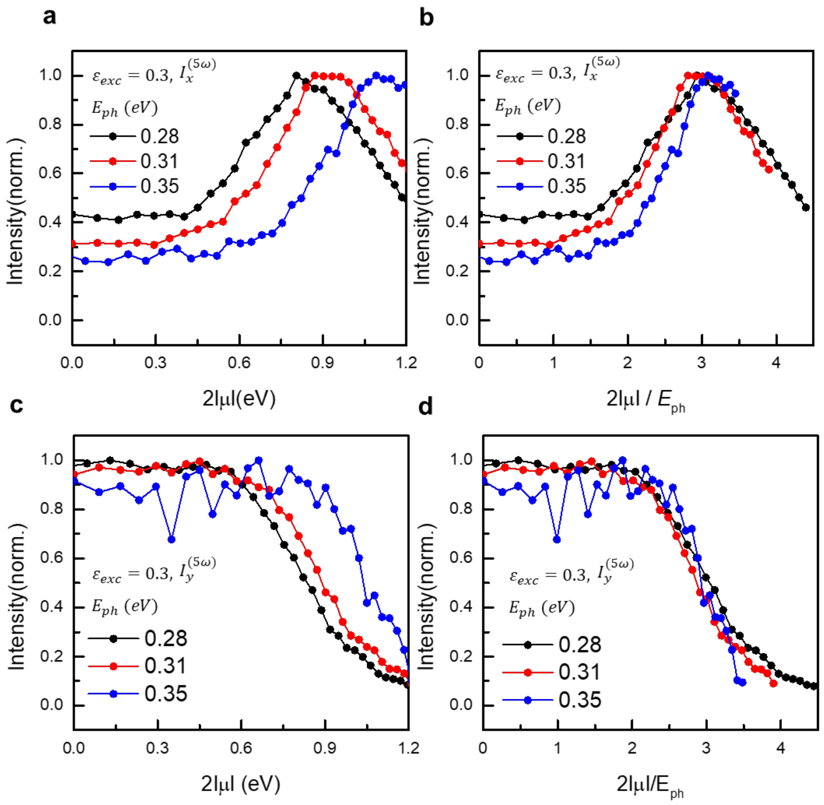

Figure. S7| μ-dependent HHG given by the elliptically-polarized excitation with various E_{ph}. a, Normalized $I_x^{(5\omega)}$ versus $2|\mu|$ under elliptically-polarized excitation (ε_{exc} = 0.3) with E_{ph} of 0.28 (black circles), 0.31 (red circles) and 0.35 eV (blue circles). **b,** Normalized $I_x^{(5\omega)}$ versus $2|\mu|/E_{ph}$ under elliptically-polarized excitation (ε_{exc} = 0.3) for different E_{ph}. **c,** Normalized $I_y^{(5\omega)}$ versus $2|\mu|$

under elliptically-polarized excitation ($\varepsilon_{\mathrm{exc}}$ = 0.3) for different E_{ph}. **d,** Normalized $I_{\mathrm{y}}^{(5\omega)}$ versus $2|\mu|/E_{\mathrm{ph}}$ under elliptically-polarized excitation ($\varepsilon_{\mathrm{exc}}$ = 0.3) for different E_{ph}.

Figure S8. Polarization-resolved fifth harmonic response with control of μ and $\varepsilon_{\mathrm{exc}}$

Fig. S8 presents the polarization-resolved HHG while modulating μ (0 ~ 1.5 eV) and $\varepsilon_{\mathrm{exc}}$ (0 ~ 0.5). For all measurements, I_{exc} and E_{ph} have kept at 3.1 GW cm^{-2} and 0.28 eV, respectively. As $2|\mu|$ increases, HHG hardly rotates with the change of $\varepsilon_{\mathrm{exc}}$. The guide for the eyes (pink solid lines) corresponds to fitting equation: $I_{\mathrm{HHG}}(\varphi) = \alpha + \beta cos^2(\varphi - \gamma)$.

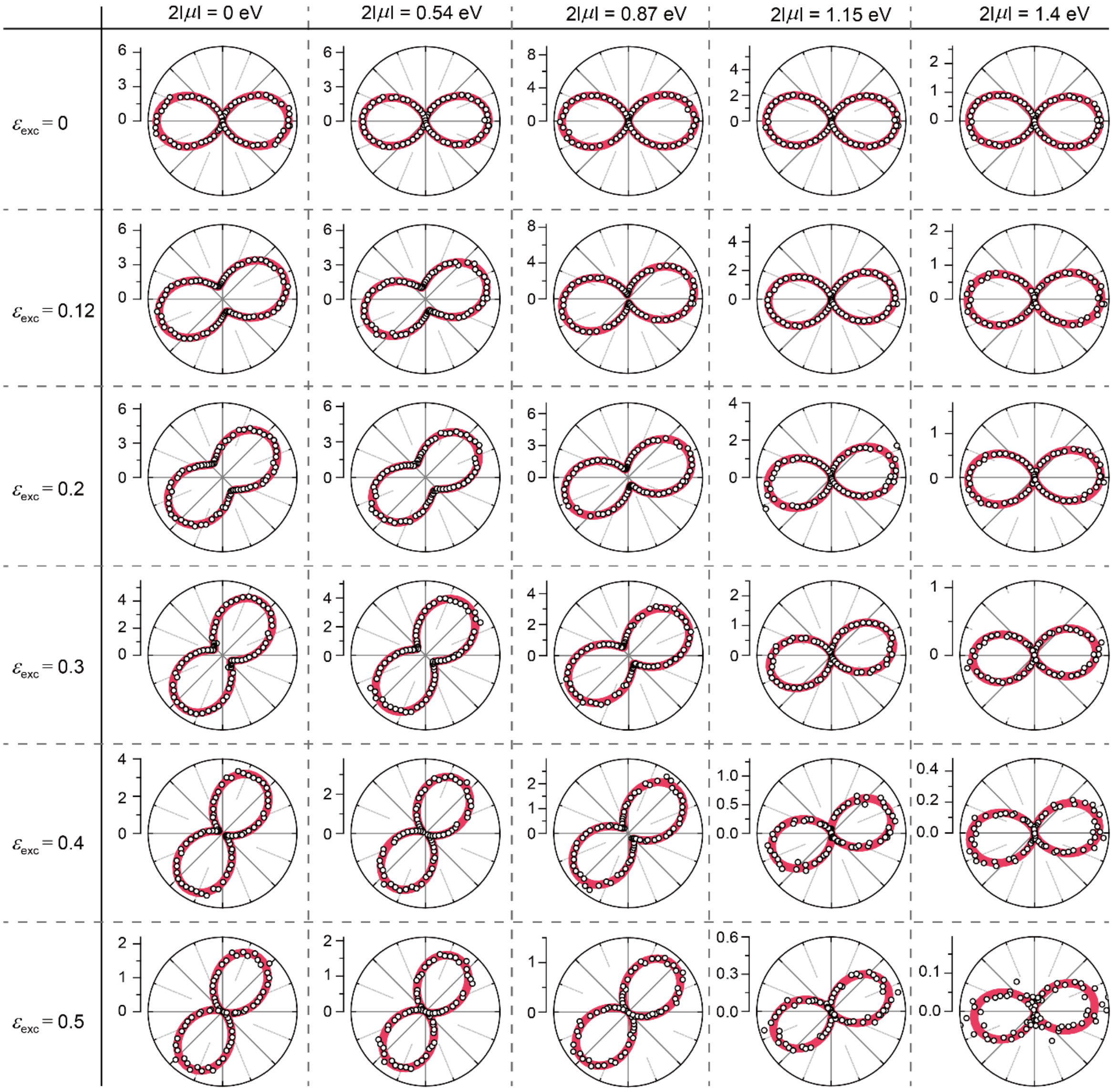

Figure. S8| Intensity profile of polarization-resolved HHG. HHG is measured under excitation laser with different $\varepsilon_{\mathrm{exc}}$ and μ. The black empty circles represent the experimental data, and the pink solid lines are guide for the eyes.

Device fabrication

Aligened polycrystalline graphene film is grown by chemical vapor deposition on Cu foil (Nilaco corporation, #CU-113213, 30 μm thick, 99.9% purity)[3]. The Cu foil is annealed at 1030 °C for 4-hour under flow of H_2 at 70 sccm with a total pressure of 5 Torr; then graphene film is grown at 1040 °C for 2-hour 30 min by additionally introducing methane in Ar carrier gas at 0.15 sccm. During the cooling process after growth, the methane injection is maintained until the film had cooled to 600 °C, then only H_2 is introduced until the temperature reached < 150 °C to allow unloading of the sample. Poly (methyl methacrylate) (PMMA, 996K, 8% in Anisole) is spun onto as grown graphene. To etch the Cu substrate, the PMMA/graphene/Cu foil is floated on a Cu etchant of $FeCl_3$ aqueous solution (Sigma-Aldrich, #667528) for 30 min. After complete etching of Cu, the PMMA/graphene film is transferred onto a surface of ultrahigh purity deionized water (DIW) by scooping with a SiO_x substrate, then and releasing the ultrathin film. The process is repeated twice in a clean DIW to rinse the surface, then the film is transferred onto a double-side polished sapphire substrate. The sample is dried by heating at 60 °C in air for 10 min, followed by annealing at 165 °C for 10 min and soaking in acetone at 60 °C for 10 min to remove the PMMA. Finally, annealing is performed at 350 °C in air for 15 min to remove the PMMA residue.

After transfer process, drain, source, and side gate contacts are deposited by e-beam evaporator (Cr 5 nm / Au 50 nm) to construct ion-gel-based field-effect transistor. For ion-gel gate dielectric, 0.1 g of poly(vinylidene fluoride-co-hexafluoropropylene) (PVDF-HFP) and 0.195 g of 1-ethyl-3-methylimidazolium bis (trifluoromethylsulfonyl)imide ([EMIM][TFSI]) are dissolved in the mixture of 2 ml of 2-butanon and 0.25 ml of propylene carbonate. [EMIM][TFSI] and PVDF-HFP were purchased from Sigma Aldrich and ARKEMA, respectively. The solution is stirred overnight at 45°C, then drop-casted onto the device to cover gate electrodes and graphene channel. The sample is dried in argon filled glove box. Source, drain, and gate contacts are electrically connected by pair of voltage source meter (Keithley 2400, 2450) to apply gate voltage and read channel resistance. During both infrared transmission and HHG measurements, gate leakage current and channel resistance are carefully monitored to ensure reproducible performance of ion-gel gate dielectric.

Infrared transmission spectroscopy

Infrared transmission spectroscopy is employed to identify the effective optical bandgap with a sweeping gate voltage[4] and thus, determine the exact relation between V_G and μ. Fig. S9 shows the experimental configuration. Infrared transmission measurement has been performed using a stabilized tungsten-halogen light source (SLS201L/M, Thorlabs). To allow the light to pass only through the graphene, the beam size is adjusted by placing an iris just before the sample. The transmission spectra

are collected by a grating spectrometer (SP-2300, Princeton Instruments) equipped with a cryogenically cooled InGaAs detector (PyLoN IR, Princeton Instruments). With the ion- gel electrostatic gating, we have characterized the chemical potential of the graphene device using infrared transmission spectroscopy. The gate leakage current and channel resistance are measured simultaneously, and reproducible transfer characteristics are recorded for multiple gate sweeps during the measurement. The V_{G} of the charge neutral case (V_{CNP}) is measured from the channel resistance curve, and the transmission spectra different V_{G} are normalized by the one gated at the V_{CNP}. The differential transmission spectrum is related to the optical conductivity of graphene through the following relation

$$\frac{T(\mathrm{V_G})}{T(\mathrm{V_{CNP}})} = = \frac{\left|\frac{1}{\eta_1}+\frac{1}{\eta_2}+\sigma_{\mathrm{V_{CNP}}}\right|^2}{\left|\frac{1}{\eta_1}+\frac{1}{\eta_2}+\sigma_{\mathrm{V_G}}\right|^2}, \tag{1}$$

where η_1 and η_2 are the wave impedances of the sapphire substrate and ion-gel, respectively. According to Kubo's formula, the optical conductivity at a frequency ω can be calculated using the following relation[5]:

$$\sigma(\omega) = \frac{1}{2}\sigma_0[\tanh\left(\frac{\hbar\omega+2|\mu|}{4k_B T_e}\right) + \tanh(\frac{\hbar\omega-2|\mu|}{4k_B T_e})], \tag{2}$$

where σ_0 is the universal optical conductivity of graphene and T_e is an electron temperature. Now, $2|\mu|$ can be extracted by fitting measured spectra using equation (1) and (2). Owing to density of states at Dirac cone, carrier concentration n can be derived using $n = \mu^2/\pi\hbar^2 v_F^2$, where v_F is the Fermi velocity. Considering both the quantum and geometrical capacitances of graphene device, chemical potential is related to the gate voltage based on following equation

$$V_{\mathrm{G}} - V_{\mathrm{CNP}} = \frac{\mu}{e} + \frac{ne}{C}, \tag{3}$$

where C is the capacitance of ion-gel dielectric, which differed by device geometry in the range of 4.8-8.9 μF cm^{-2}. Extracted chemical potential fits well for multiple devices. This infrared transmission spectroscopy has been performed before and after the HHG measurement to ensure stability of ion-gel-based field-effect transistor.

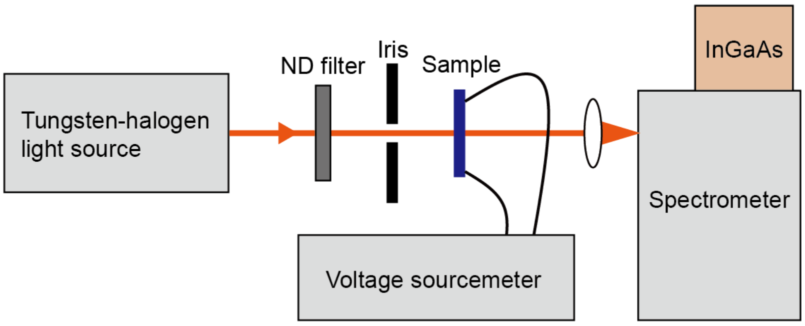

Figure S9| Schematic of the experimental setup for the infrared transmission measurement

HHG measurements

Fig. S10 illustrates the experimental setup used for the HHG measurements. A femtosecond laser system (Light Conversion PHAROS), mid-infrared pulses are prepared using optical parametric amplifier (ORPHEUS) and a difference frequency generator (LYRA). The output serves wavelength-tunable multi-cycle pulses with repetition rate of 100 kHz. The spectral linewidth of pulse is 15.4 meV (full-width at half-maximum), and the pulse duration is estimated to be 120 fs assuming a Fourier-transform-limited pulse. To control its ellipticity, a liquid crystal retarder (Thorlabs LCC1111-MIR) is employed, whose optical axis is oriented at an angle of 45 degrees with respect to the laser polarization. Then, the mid-infrared pulses are focused on the graphene device using ZnSe focusing objectives. The emitted HHG has been collected using 50X objective lens on transmission geometry, and the HHG polarization is analyzed by half-wave plate mounted on motorized stage and fixed Glan-Taylor polarizer. The HHG spectra are recorded by an electron-multiplying charge-coupled device detector (ProEM, Princeton instruments) and a grating spectrometer (SP-2300, Princeton instruments).

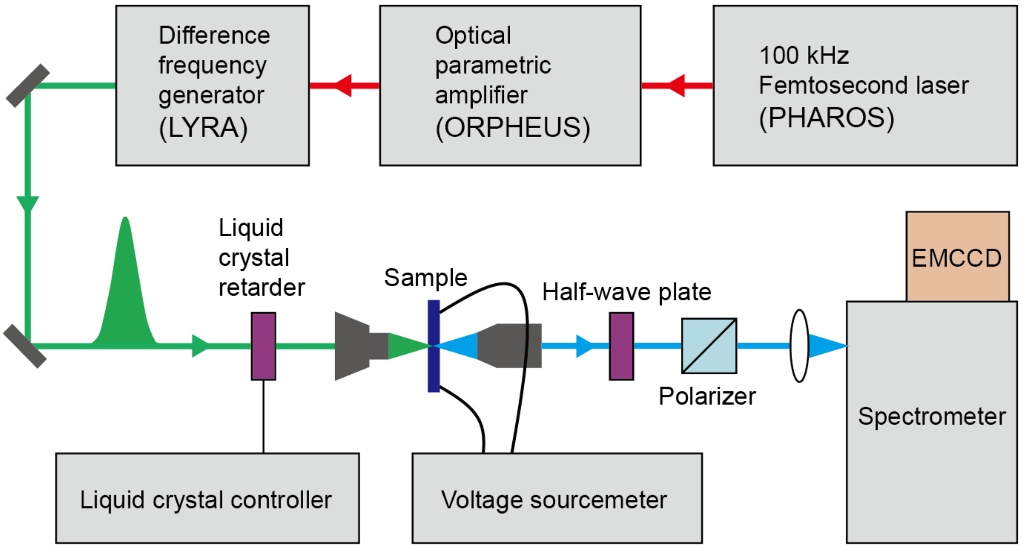

Figure. S10| Schematic of the experimental setup for the HHG measurement.

Transition dipole moment of massless Dirac fermions

Hamiltonian H_0 of graphene can be written by the following near two equivalent Dirac points:

$$H_0 = \pm v_F \sigma \cdot \mathbf{k}$$

where v_F is Fermi velocity of graphene, $\sigma = (\sigma_x, \sigma_y)$ are the Pauli matrices, and $\mathbf{k}$ is the Bloch wave vector. + and – signs correspond to the conduction and valence bands of graphene, respectively.

The Hamiltonian under external laser field is modified as following equation:

$H = \pm v_F \sigma \cdot (\mathbf{k} + \mathbf{A}) = \pm v_F (\sigma \cdot \mathbf{k} + \sigma \cdot \mathbf{A}) = H_0 + H'$,

where H' corresponds to interaction with laser field H'. Optical transition rate is determined by following:

$$\langle c|H'|v\rangle \sim \langle c|\sigma \cdot \mathbf{A}|v\rangle = \langle c|\sigma|v\rangle \cdot \mathbf{A}$$

Therefore, transition dipole moment of graphene is proportional to $\langle c|\sigma|v\rangle$ which is winding around the Dirac point as shown in Fig. 4c. We note that similar transition dipole moment profile exists in other topological semimetals including topological insulators and Weyl semimetals where hamiltonian can be written in the form of $\sigma \cdot \mathbf{k}$.

Quantum master equation

To demonstrate our experimental HHG results, we employee the quantum master equation widely used to solve density matrices differential equation based on the Houston basis[6] reads,

$$\frac{\partial}{\partial\tau}\rho_{\mathbf{k}}(\tau) = -i\left[H_{\mathbf{k}+\frac{\mathbf{A}(\tau)}{c}}, \rho_{\boldsymbol{k}}(\tau)\right] + D_e[\rho_{\boldsymbol{k}}(\tau)], \tag{4}$$

Unless otherwise noted, hereafter we define the dephasing operator as $D_e[\rho_{\boldsymbol{k}}(\tau)] := -(1-\delta_{nm})\rho_{\boldsymbol{k}}^{nm}(\tau)/T_2$, and $\mathbf{A}(\tau)$ the time-dependent external vector potential. The Hamiltonian contains Dirac cone features where the $\mathbf{k}$ is expanded from the momentum K and K', which can be written as,

$$H_{\mathbf{k}+\frac{\mathbf{A}(\tau)}{c}} = v\chi\sigma_x\left(k_x + \frac{A_x(\tau)}{c}\right) + v\sigma_y\left(k_y + \frac{A_y(\tau)}{c}\right), \tag{5}$$

The χ and v denote a symmetry parameter (1 or -1) for each Dirac cone vertices and the Fermi velocity of 0.51 atomic unit, respectively. Finally, we obtain HHG intensity of arbitrary frequency ω derived from the real-time current density, $I(\omega) = \omega^2\left|\int d\tau e^{i\omega\tau} cTr\left[\frac{\partial H_{\mathbf{k}+\frac{\mathbf{A}(\tau)}{c}}}{\partial\mathbf{A}(\tau)}\rho_{\boldsymbol{k}}(\tau)\right]\right|^2$ and integrated intensities to define HHG order, $I(n) = \int_{n\omega_{exc}-0.5\omega_{exc}}^{n\omega_{exc}+0.5\omega_{exc}} d\omega I(\omega)$. The estimated fifth order HHG spectra are displayed in the Fig. S11. When linearly polarized (x-polarized, $\varepsilon_{\mathrm{exc}} = 0$) pump is applied to the graphene, only x-polarized HHG signal can be generated. If a pump pulse with higher ellipticity $\varepsilon_{\mathrm{exc}} > 0$ excites the Dirac electrons, then y-polarized HHG signal is significantly enhanced. Considering the dielectric function of the graphene surrounded by the ion-gels, the peak electric field strength of the time-dependent electric field, i.e., $\mathbf{E}(\tau) = -\frac{1}{c}\frac{\partial\mathbf{A}(\tau)}{\partial\tau}$, is adopted to be 8.5 meV Å^{-1} which corresponds to the 3.1 GW cm^{-2} of experiment.

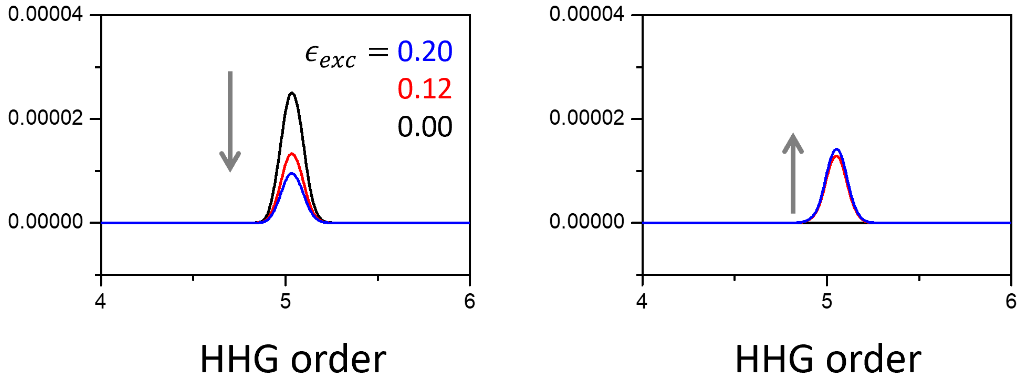

Figure. S11| Fifth order HHG intensity with respect to various ellipticities 0, 0.12, and 0.2. The left and right panels denote for x- and y-polarized HHG signals, respectively. To estimate the arbitrarily

polarized (from x to x' or cos$\theta = \frac{\hat{\mathbf{x}}^{'} \cdot \hat{\mathbf{x}}}{|\hat{\mathbf{x}}'||\hat{\mathbf{x}}|}$) HHG intensity, one can evolve the rotation transform of J_x and J_y into the new axes of *x'* and *y'* , that is, $J_{\mathrm{x}'}(\tau,\theta) = J_{\mathrm{x}}(\tau)\cos\theta + J_{\mathrm{y}}(\tau)\sin\theta$, finally the polarization rotated HHG intensity can be given as $I(n,\theta)$. Here the θ is defined as an angle between *x* and *x'*.

Liouville-von Neumann equation

We need to explore behind interplays between the inter-band and intra-band excitation channels induced from an external vector potential. Employing the Liouville-von Neumann equation with velocity gauge space within the Bloch states as a basis of fixed momentum, it may deliver clear insights to divide the inter and intra band transitions of the Dirac electrons. The external vector potential can be decomposed into the inter- and intra-band excitation channels through, which reads,

$$H_{\boldsymbol{k}}(\tau) = H_0(\mathbf{k}) + \mathbf{p} \cdot \frac{\mathbf{A}(\tau)}{c}, \tag{6}$$

The H_0 provides diagonalized field-free graphene Dirac bands and the $\mathbf{p}$ denotes the inter- and intra-band momentum matrix. Then the optical interaction term can be obtained,

$$\mathbf{p} \cdot \frac{\mathbf{A}(\tau)}{c} = \frac{1}{c}\begin{pmatrix} p_{cc_x}A_{cc_x}(\tau) & p_{cv_x}A_{cv_x}(\tau) \\ p_{vc_x}A_{vc_x}(\tau) & p_{vv_x}A_{vv_x}(\tau) \end{pmatrix} + \frac{1}{c}\begin{pmatrix} p_{cc_y}A_{cc_y}(\tau) & p_{cv_y}A_{cv_y}(\tau) \\ p_{vc_y}A_{vc_y}(\tau) & p_{vv_y}A_{vv_y}(\tau) \end{pmatrix} \tag{7}$$

From the decomposed gauge field, we can handle the model gauge that can be used to discover the excitation physics from several arbitrarily independent channels including only intra-band excitation, only inter-band excitation, and inter- intra- paired excitations with their own directions, i.e., x and y, by controlling each parameter of $A_{ij_\eta}(\tau)$. The diagonal parts and off-diagonal parts belong to the $M_{x,y}^{intra}$ and $M_{x,y}^{inter}$, respectively. The optical transition matrix elements $\mathbf{p}$ can be obtained by the inter- and intra-band momentum matrix with the field-free eigen states at the given momentum $\mathbf{k}$, i.e., $p_{ij_\eta} = \langle i\mathbf{k}|\frac{\partial}{\partial \mathbf{k}}H_0(\mathbf{k})\hat{\eta}|j\mathbf{k}\rangle$. The $|i\mathbf{k}\rangle$ is the eigen vector corresponding to the Hamiltonian $H_0(\mathbf{k})$.

Dephasing parameter

From the eq. (4), we need to define the dephasing time of T_2 corresponding to the experimental results. We find that the rotated angle of the major axis of HHG ellipse is found to have a sensitive response with respect to the dephasing time, T_2. In experiment, the angle of the major axis is observed to be $21°$ for $\varepsilon_{\rm exc}$ of 0.12 so that we can explicitly choose proper T_2 to be ~ 4 fs to make consistence with theoretical calculations.

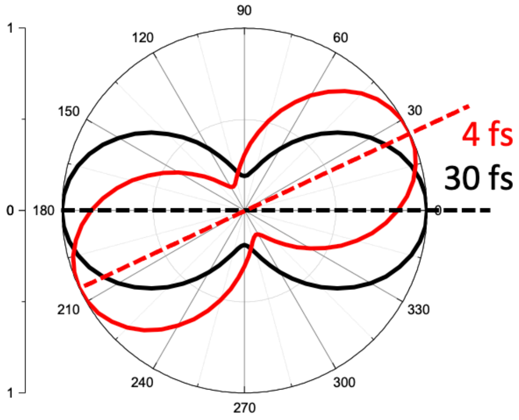

Figure. S12| Calculated normalized polar plot of HHG and the rotation angle of the major axis with respect to the dephasing time of 4 fs and 30 fs with $\boldsymbol{\varepsilon_{\rm exc} = 0.12}$.

Calculation of chemical potential dependent HHG

Employing the quantum master equation, we simulate HHG as a function of various chemical potential. When the applying laser pulse is linearly polarized (left panel), only I_x is found with a strong peak around at the $2|\mu|$ of 0.94 eV which monotonically decrease at the higher photon regime. As the ellipticity increases, the I_y starts to increase rapidly and exceeds the I_x (middle and right panels).

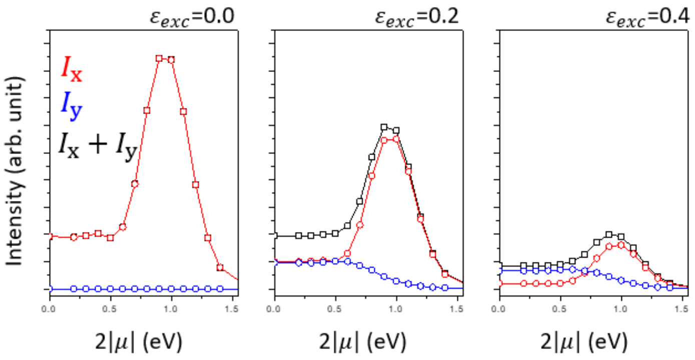

Figure. S13| Calculated HHG intensities as a function of chemical potential.

***I*_exc - dependence of the fifth harmonic expected from perturbative nonlinear optics**

Under elliptically-polarized excitation, the polarization axis of the 5th harmonics rotates as the laser intensity increases from 2.4 GW cm^{-2} to 10.1 GW cm^{-2} while the ellipticity of the laser pulse is fixed at 0.3, as shown in Fig. 1g. This rotation of the polarization axis is clear indication of the non-perturbative response at 3.1 GW cm^{-2}, since the polarization axis cannot rotate as the intensity increases in nonlinear optics in the perturbative regime, where the fifth-order susceptibility are independent on the laser intensity. In the perturbative regime, the intensity of a fifth harmonic signal $I_i(5\omega)$ under laser-field $\mathbf{E}(\omega)$ can generally be calculated by the following equation

$$I_i(5\omega) \propto \left|P_i^{(5)}(5\omega)\right|^2 \propto \left|\sum_{jklmn} \chi_{ijklmn}^{(5)}(5\omega)E_j(\omega)E_k(\omega)E_l(\omega)E_m(\omega)E_n(\omega)\right|^2 \quad (8)$$

where $P_i^{(5)}(5\omega)$ is a induced nonlinear polarization at 5ω and $\chi_{ijklmn}^{(5)}$ is the component of fifth-order nonlinear susceptibility tensor. In our HHG set up in the transmission geometry, E_z becomes zero, which simplifies all components as follows

$$\begin{pmatrix} \widetilde{P_x} \\ \widetilde{P_y} \end{pmatrix} = \begin{pmatrix} \chi_{xxxxxx} & \chi_{xyyyyy} & \chi_{xxyyyy} & \chi_{xxxyyy} & \chi_{xxxxyy} & \chi_{xxxxxy} \\ \chi_{yxxxxx} & \chi_{yyyyyy} & \chi_{yxyyyy} & \chi_{yxxyyy} & \chi_{yxxxyy} & \chi_{yxxxxy} \end{pmatrix} \begin{pmatrix} E_x^5 \\ E_y^5 \\ E_x E_y^4 \\ E_x^2 E_y^3 \\ E_x^3 E_y^2 \\ E_x^4 E_y \end{pmatrix}. \quad (9)$$

$\widetilde{P_x}$ and $\widetilde{P_y}$ are the fifth harmonic polarization along x-axis and y-axis, respectively, which can be expressed with the following equations

$$\widetilde{P_x} = P_x e^{i\delta_x} \quad (10)$$

$$\widetilde{P_y} = P_y e^{i\delta_y} \quad (11)$$

where P_x (P_y) and δ_x (δ_y) are magnitude and phase of the polarization along x-axis (y-axis), respectively. In the case of the laser excitation of ellipticity ϵ, E_y can be replaced to $(i\epsilon)E_x$

$$\begin{pmatrix} \widetilde{P_x} \\ \widetilde{P_y} \end{pmatrix} = \begin{pmatrix} \chi_{xxxxxx} & \chi_{xyyyyy} & \chi_{xxyyyy} & \chi_{xxxyyy} & \chi_{xxxxyy} & \chi_{xxxxxy} \\ \chi_{yxxxxx} & \chi_{yyyyyy} & \chi_{yxyyyy} & \chi_{yxxyyy} & \chi_{yxxxyy} & \chi_{yxxxxy} \end{pmatrix} \begin{pmatrix} E_x^5 \\ (i\epsilon)^5 E_x^5 \\ (i\epsilon)^4 E_x^5 \\ (i\epsilon)^3 E_x^5 \\ (i\epsilon)^2 E_x^5 \\ (i\epsilon)^1 E_x^5 \end{pmatrix} \quad (12)$$

$\widetilde{P_x}$ and $\widetilde{P_y}$ are represented by tensor components and E_x

$$\widetilde{P_x} = (\chi_{xxxxxxx} + \chi_{xyyyyyy}(i\epsilon)^5 + \chi_{xxyyyyy}(i\epsilon)^4 + \chi_{xxxxyyy}(i\epsilon)^3 + \chi_{xxxxxyy}(i\epsilon)^2 + \chi_{xxxxxxy}(i\epsilon))E_x^5$$
(13)

$$\widetilde{P_y} = \left(\chi_{yxxxxxx} + \chi_{yyyyyyy}(i\epsilon)^5 + \chi_{yxyyyyy}(i\epsilon)^4 + \chi_{yxxxyyy}(i\epsilon)^3 + \chi_{yxxxxyy}(i\epsilon)^2 + \chi_{yxxxxxy}(i\epsilon)\right) E_x^5 \ .$$
(14)

Therefore, both relative magnitude and phase of $\widetilde{P_x}$ and $\widetilde{P_y}$ do not change over laser intensity. For elliptical polarization, angle of the major polarization axis θ can be expressed with P_x, P_y, and relative phase δ between P_x and P_y ($\delta = \delta_x - \delta_y$)[7].

$$\tan 2\theta = \frac{2P_xP_y cos\delta}{(P_x)^2 - (P_y)^2} = \frac{2(P_x/P_y)cos\delta}{(P_x/P_y)^2 - 1} \qquad (15)$$

Thus, this indicates that the polarization axis of high harmonics cannot rotate over intensity in the perturbative regime.

Discussion on detailed physical origin and features of μ-dependent HHG

1) Perturbative theoretical calculation of $\chi^{(5)}$ without considering scattering process

In the perturbative regime without considering the scattering process, μ-dependent HHG shows series of multiple sharp resonance-like profiles at $2|\mu|/E_{ph}$ = n (n is an integer). Such profile has been theoretically predicted for $I_x^{5\omega}$ by the recent theoretical study[8] and Fig. S14 describes μ-dependent fifth-order susceptibility $\chi^{(5)}$ with following equation

$$\sigma^{(5)}(\omega,\mu) = \frac{9i\sigma_5}{655360\pi}\left(\frac{2v_F}{3a_0\omega}\right)^8 \left[4925\ln\frac{2\mu-5\omega}{2\mu+5\omega} - 16384\ln\frac{2\mu-4\omega}{2\mu+4\omega} + 19359\ln\frac{2\mu-3\omega}{2\mu+3\omega} - 9216\ln\frac{2\mu-2\omega}{2\mu+2\omega} + 1266\ln\frac{2\mu-\omega}{2\mu+\omega}\right]$$

$$\propto i5\omega\chi^{(5)}(\omega,\mu) \tag{16}$$

where $\sigma^{(5)}(\omega)$ is the fifth-order conductivity of graphene, σ_5 is fifth-order 2D conductivity, v_F is the Fermi velocity, a_0 is the distance between carbon atoms, and ω is frequency of the excitation laser.

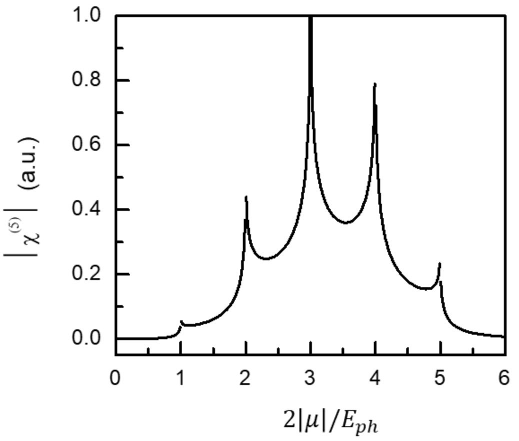

Figure. S14| Perturbative theoretical calculation of $\chi^{(5)}$ without considering scattering process. The fifth harmonic susceptibility of graphene $\chi^{(5)}$ versus $2|\mu|/E_{ph}$ is calculated without any broadening contributions, exhibiting series of sharp resonance-like profiles at $2|\mu|/E_{ph}$ = *n*.

2) Quantum master equation calculation of the fifth harmonic intensity and current

μ-dependent HHG might show series of multiple sharp resonance-like profiles without considering scattering process. However, intense laser excitation creates high density of photocarriers in graphene, which enables rapid electron scattering process. Then, series of sharp resonance-like profiles can be drastically broadened and merged together, forming one resonance-like profile located at $2|\mu|/E_{ph}$ = non-integer. In order to investigate such possibility for $I_x^{5\omega}$ in our case, we have employed quantum master equation to theoretically calculate 5th harmonic intensity as a function of chemical potential (Fig. S15a) which fully takes into account electronic scattering processes under strong laser field. Fig. S15b and S15c show how massless Dirac states around the Dirac point in the momentum space contributes to generate 5th harmonic current $J_x^{5\omega}$ in graphene under linearly polarized excitation along the x-direction (E_x). Dashed lines are constant energy contours on a Dirac cone, which describes electronic states vertically separated by energy mE_{ph}. m is an integer. Real and imaginary parts of $J_x^{5\omega}$ (Fig. S15b and S15c, respectively) are most strongly generated from Dirac states in the region between $2E_{ph}$ and $4E_{ph}$. Depending on the photon number required for multi-photon transitions and the azimuthal angle, Dirac states generate $J_x^{5\omega}$ with characteristic sign and magnitude, which destructively interfere when radiating $I_x^{5\omega}$. As $2|\mu|$ increases, Pauli blocking sequentially disables contribution from the Dirac states starting from near the Dirac point to higher energy. As shown in Fig. S15a, $I_x^{5\omega}$ increases as the destructive interference is partly eliminated while $I_x^{5\omega}$ eventually disappears as all the resonant Dirac states are disabled. Our theoretical calculation (Fig. R5a) also shows that the maximum intensity is exhibited around at $2|\mu|/E_{ph}$= 3.4, which is the most common value for $2|\mu|/E_{ph}$ in our experimental data (Fig.S5).

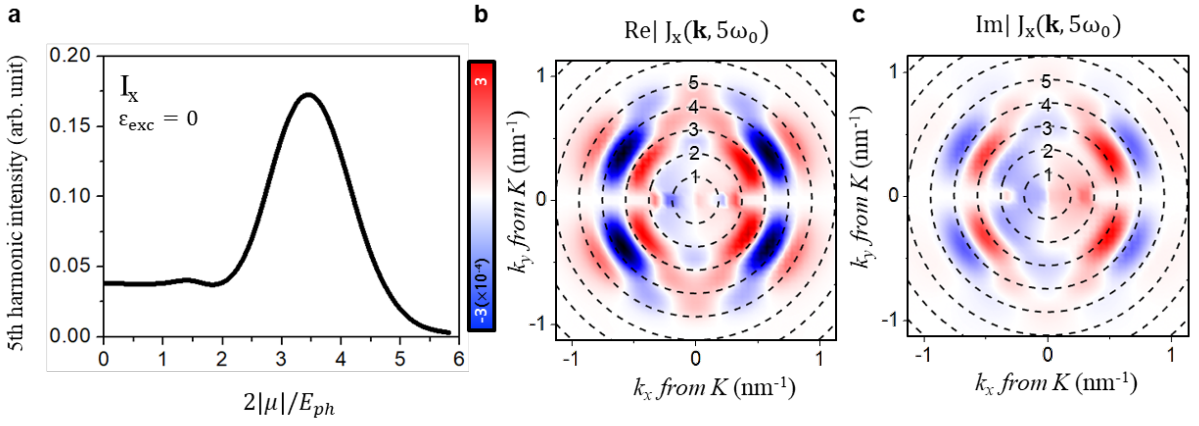

Figure. S15| Theoretical calculation of the fifth harmonic intensity and current in the momentum space. a, Calculated fifth harmonic intensity as a function of $2|\mu|/E_{ph}$ under linearly-polarized excitation with I_{exc} of 3.1 GW cm^{-2}. **b and c,** (b) Real part and (c) imaginary part of 5th harmonic current $J_x^{5\omega}$ generated by Dirac states around Dirac point in the momentum space. The red (blue) color represents for positive (negative) values. Dashed lines are constant energy contours on a Dirac cone,

which describe the conduction band states vertically separated by nE_{ph} from the valence band.

3) Variation of the detailed characteristics over different devices

Although overall profiles of $I_x^{5\omega}$ as a function of $2|\mu|$ are similar, detailed features vary over different devices. $I_x^{5\omega}$ exhibits notable dip-like response around $2|\mu|$ = 0.4 eV in Fig.2c while $2|\mu|$ for the maximum $I_x^{5\omega}$ varies from 0.87 eV and 0.99 eV. It implies that quality of ion-gel or graphene grown by chemical vapor deposition (CVD) might affect the detailed features in the curve of $I_x^{5\omega}$ versus chemical potential. Also, humidity fluctuation can cause experimental uncertainty in terms of determination of the exact chemical potentials versus gate voltages.

To further investigate the origin of their variations, we calculated the μ-dependence of $I_x^{5\omega}$ for varying dephasing lifetime based on quantum master equation. Interestingly, our theoretical calculation (Fig. S16b) also shows a dip-like profile around at $2|\mu|$ = 0.4 eV. As dephasing time T_2 decreases from 4 fs to 2 fs, the dip-like profile becomes more pronounced while the resonance-like profile (originating from channels via multiphoton transitions) is heavily suppressed. This suggests that there exist other channels which generates $I_x^{5\omega}$ without going through multiphoton transitions. Identifying the physical origin of this channel requires extensive experimental and theoretical investigation with systematic control of laser intensity and sample qualities which will allow quantify and control T_2.

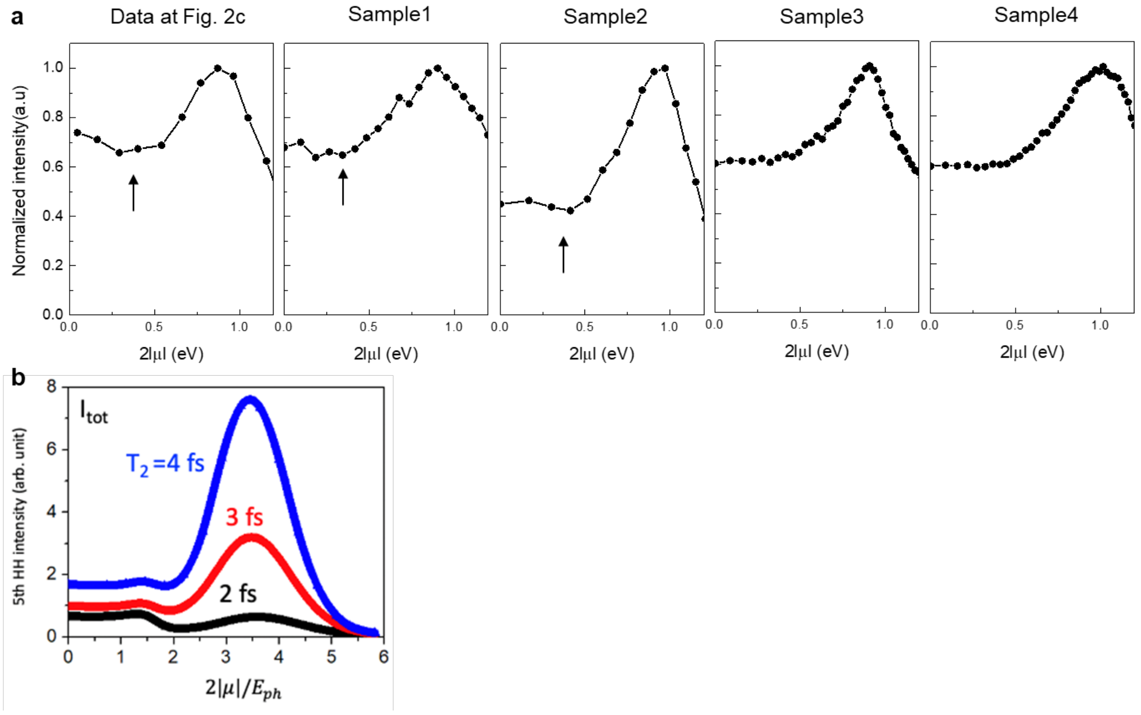

Figure. S16| μ-dependent HHG measured at multiple samples and given by theoretical calculation. a, Normalized harmonic intensity versus $2|\mu|$ taken from four different samples under

linearly-polarized excitation with E_{ph} of 0.28 eV. All data are acquired under same experimental conditions as the measurement for Fig. 2c. The dip-like profiles around $2|\mu|$ = 0.4 eV shown in Fig. 2c (marked with black arrow) is observed in sample 1 and 2, but not in sample 3 and 4. **b,** Harmonic intensity is calculated with T_2 of 4 fs (blue curve), 3 fs (red curve) and 2 fs (black curve).

4) Contribution from one-photon interband transition to HHG

Although one photon transition does excite tremendous number of charge carriers, which can be blocked when $2|\mu|/E_{\mathrm{ph}}= 1$. However, only small fraction of these photo-excited carriers participates to generate 5th harmonics through series of interband or intraband transitions across all electronic states in the momentum space. Instead, carrier excitation via multi-photon transitions can provide more efficient pathways to generate 5th harmonics as shown in Fig.S14. In the perturbation regime, Fig. S14 shows susceptibility for 5th harmonics generation as a function of chemical potential. Contribution from one photon transition is drastically weaker than three- or four-photon transitions.

Furthermore, as schematically shown in Fig. S17, our mid-infrared excitation laser has many-cycles of laser-field. Before reaching sufficiently strong laser-field in the middle of the pulse, where supposedly the 5th harmonic is most strongly generated, first few cycles of laser-field can create large number of background photocarriers which effectively increase doping in graphene even when “static” chemical potential is placed at the Dirac point. For this reason, we believe that we do not observe any abrupt change at $2|\mu|/E_{\mathrm{ph}}$ = 1.

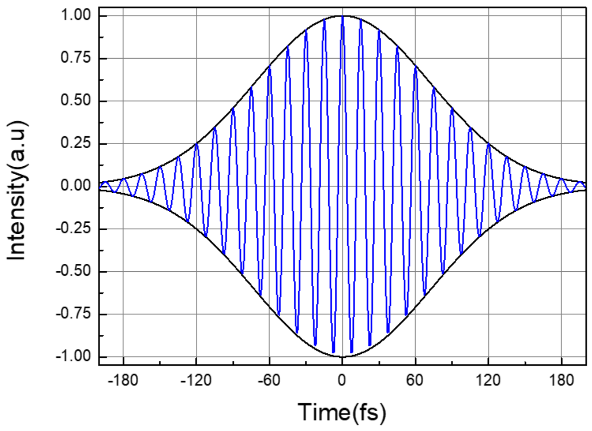

Figure. S17| Temporal profile of the laser pulse employed as excitation source. Estimated temporal profile of electric field for excitation laser pulse. Difference frequency generation serves multi-cycle mid-infrared pulses with pulse width of 120 fs and E_{ph} of 0.28 eV.

Supplementary Note

References

1 Yoshikawa, N., Tamaya, T. & Tanaka, K. High-harmonic generation in graphene enhanced by elliptically polarized light excitation. *Science* **356**, 736-738 (2017).

2 Kim, Y. *et al*. Bright visible light emission from graphene. *Nat. Nanotechnol.* **10**, 676-681 (2015).

3 Brown, L. *et al*. Polycrystalline graphene with single crystalline electronic structure. *Nano Lett.* **14**, 5706-5711 (2014).

4 Chen, C. –F. *et al*. Controlling inelastic light scattering quantum pathways in graphene. *Nature* **471**, 617-620 (2011).

5 Stauber, T., Peres, N. M. R. & Geim, A. K. Optical conductivity of graphene in the visible region of the spectrum. *Phys. Rev. B* **78**, 085432 (2008).

6 Sato, S. A., Hirori, H., Sanari, Y., Kanemitsu, Y. & Rubio, A. High-order harmonic generation in graphene: Nonlinear coupling of intraband and interband transitions. *Phys. Rev. B* **103**, L041408 (2021).

7 Hecht, E. *Optics* (Pearson, 2017).

8 Hipolito, F., Dimitrovski, D. & Pedersen, T. G. Iterative approach to arbitrary nonlinear optical response functions of graphene. *Phys. Rev. B* **99**, 195407 (2019).
